# Choroidal circulation disturbance is an initial factor in outer retinal degeneration in rats under simulated weightlessness

**DOI:** 10.3389/fphys.2023.1198862

**Published:** 2023-07-20

**Authors:** Yuxue Mu, Dongyu Wei, Lilingxuan Yao, Xinyue Xu, Shaoheng Li, Ruidan Cao, Tao Chen, Zuoming Zhang

**Affiliations:** ^1^ Aerospace Clinical Medical Center, School of Aerospace Medicine, Air Force Medical University, Xi’an, China; ^2^ Department of Aviation Medicine, Xijing Hospital, Air Force Medical University, Xi’an, China; ^3^ The Third Regiment, School of Basic Medicine, Air Force Medical University, Xi’an, China

**Keywords:** simulated weightlessness, outer blood-retinal barrier, retinal degeneration, choroid, Cx43, ZO-1

## Abstract

**Objective:** Microgravity contributes to ocular injury yet the underlying mechanism remains unclear. This study aims to elucidate the mechanism behind choroidal circulation disorder and outer retinal degeneration in rats with simulated weightlessness.

**Methods:** Optical coherence tomography angiography (OCTA) was used to evaluate choroidal circulation and retinal morphological alterations in rats with weightlessness simulation. Electroretinogram and transmission electron microscopy were used to examine the ultrastructure and function of the choroid and outer retina. Furthermore, histological and terminal deoxynucleotidyl transferase deoxyuridine dUTP nick-end labeling (TUNEL) staining was used to monitor retinal morphology. Western blotting was performed to analyze the expressions of blood-retinal outer barrier function-related proteins (Cx43, ZO-1, and occludin).

**Results:** The choroidal thickening was observed from the fourth week of simulated weightlessness (*p* < 0.05), and choroidal capillary density started to decline by the fifth week (*p* < 0.05). Transmission electron microscopy revealed that the choroidal vessels were open and operating well by the fourth week. However, most of the mitochondria within the vascular endothelium underwent mild swelling, and by the fifth week, the choroidal vessels had various degrees of erythrocyte aggregation, mitochondrial swelling, and apoptosis. Additionally, ERG demonstrated a decline in retinal function beginning in the fifth week (*p* < 0.05). TUNEL staining revealed a significantly higher apoptotic index in the outer nuclear layer of the retina (*p* < 0.05). At the sixth week weeks of simulated weightlessness, OCTA and hematoxylin and eosin (HE) staining of retinal sections revealed that the outer nuclear layer of the retina started to become thin (*p* < 0.05). Results from western blotting revealed that Cx43, ZO-1, and occludin exhibited decreased expression (*p* < 0.05).

**Conclusion:** Based on our findings in a rat model of simulated weightlessness, choroidal circulation disturbance induced by choroidal congestion is the initial cause of outer retinal degeneration. Blood-retinal barrier disruption is significant in this process.

## 1 Introduction

With the increasing growth of space program, long stays in space will become normal for astronauts. The microgravity environment has become one of the major issues affecting astronauts’ health during flight (Prasad et al., 2020). Microgravity environment can lead to bone loss (Stein, 2013), neurological dysfunction (Roberts et al., 2017), abnormal cardiovascular function (Vernice et al., 2020), immune system function (Sun et al., 2021), and changes in the metabolism and nutritional status of astronauts (Dickerson et al., 2023). The eye is very sensitive to microgravity (Corydon et al., 2016). After entering the microgravity environment, astronauts can experience various eye changes. These manifest primarily as unilateral retinal nerve fiber layer thickening, bilateral optic disc edema, globe flattening, choroidal and retinal folds, hyperopic refractive error shifts, and local retinal ischemia (i.e., cotton wool spots). The National Aeronautics and Space Administration (NASA) defined these changes as a spaceflight-associated neuro-ocular syndrome (SANS) (Mader et al., 2019; Martin Paez et al., 2020). The mechanism underlying the eye changes during weightlessness is still incompletely understood. These changes may be caused by a corresponding increase in the blood supply to the eyes during weightlessness in space flight, arterial constriction, venous congestion, and a mismatch between intraocular and intracranial pressure. Moreover, it is also related to radiation, high concentrations of CO_2_, nutritional factors, and genetics (Zhang and Hargens, 2018; Mao et al., 2019; Yatagai et al., 2019). Without timely intervention or treatment, these pathological alterations brought on by a weightless environment may not only hinder the execution of flight missions but also endanger astronauts’ safety. Therefore, examining the effects and damage of a microgravity environment on the eyes has become an urgent research issue.

Continuous tracking research demonstrated that approximately 23% of short-term mission astronauts and 53% of long-term international space station (ISS) mission astronauts exhibit visual functional changes (Yang et al., 2022). Mader and coworkers reported novel findings of choroidal folds and new onset unilateral optic disc edema in an astronaut after repeating long-duration spaceflight (LDSF) missions. The new onset of optic disc edema after repeating LDSF nearly a decade later suggests a longstanding and possibly permanent effect of spaceflight on the neuro-ocular system that predisposes repeat flyers to more severe SANS (Huang et al., 2019; Mader et al., 2020). For this reason, we make assumptions and conjectures based on relevant research. The impact of space microgravity environment on astronauts is likely related to flight time, and a long flight mission may cause more serious damage to their health.

The SANS is associated with the headward fluid shifts incurred in microgravity during long-duration missions (Khossravi and Hargens, 2021). Current research shows that when humans are in a microgravity environment, the most significant change *in vivo* is a cephalad fluid shift as the pressure and area of the internal jugular vein increase and flow patterns change. When cerebral venous congestion affects the external scleral venous pressure and causes an increase in intraocular pressure (IOP), it also impedes the outflow of vortic veins and results in choroidal thickening. Thickening of the choroid at the back of the eye may result in foveal displacement, resulting in farsighted migration and increased intraocular pressure (IOP) (Mader et al., 1993; Huang et al., 2019). Moreover, studies have demonstrated a correlation between outer retinal thickness and choroidal oxygenation level (Kamata et al., 2022).

The choroid is among the most abundant blood-circulating tissues in the human body, and choroid circulation accounts for more than 2/3 of the eyeball’s blood flow. Each choroidal vascular layer, especially choroidal capillaries, provides oxygen and nutrients to the outer retina to maintain the hypermetabolic state of retinal photoreceptor cells. The outer blood-retina barrier, composed of choroidal capillary endothelial cells, retinal pigment epithelium (RPE), and their tight junctions, is essential for visual function (Nelson et al., 2014; Reiner et al., 2018). There is no direct evidence to prove whether the redistribution of body fluids in a microgravity environment causes choroidal microcirculation injury.

Presently, research methods for ground simulation of weightlessness mainly include the head-down-tilt bed rest for humans and the tail suspension experiment for animals (Zhang et al., 2022). The effects of the spaceflight environment on the cardiovascular, musculoskeletal, immunological, neurological, renal, metabolic, reproductive, and visual systems have been confirmed by simulated microgravity (Wise et al., 2005; Hu et al., 2017; Horie et al., 2018; Li et al., 2022; Zong et al., 2022). Animal earth-based analogs are widely used to investigate disorders that might occur during space missions. This study detected the effects of simulated microgravity on choroidal microcirculation in rodents. Moreover, the mechanism of degenerative changes in the outer retina was explained in terms of outer blood-retinal barrier function and outer retinal damage in mice with simulated weight loss.

## 2 Materials and methods

### 2.1 Animals

Male Sprague-Dawley rats (200 ± 20 g, 8 weeks of age) were provided by the Laboratory Animal Center of Air Force Medical University (Xi’an, China) (license number: 20220901). All animal experiments were approved by the Experimental Animal Ethics Committee of the Air Force Medical University, and the experimental animals were kept in accordance with the specifications for the use of scientific research animals established by the Association for Research in Vision and Ophthalmology. The rats were kept in an animal room for 1 week (12-h light and dark cycle, with free access to food and water). The temperature in the animal room was maintained at (23 ± 1)°C, at a relative humidity of approximately 50%. After 1 week, retinal function was assessed by visual electrophysiology, and rats with abnormal retinal function were excluded from the study. The experimental rats were divided into 10 groups (6 rats/group): tail suspension for 4 weeks (TS4W); tail suspension for 5 weeks (TS5W); tail suspension for 6 weeks (TS6W); tail suspension for 7 weeks (TS7W). The tail was suspended for 8 weeks (TS8W), and five controls were set at each time point.

### 2.2 Establishment of the simulated weightlessness rat model

**FIGURE 1:**
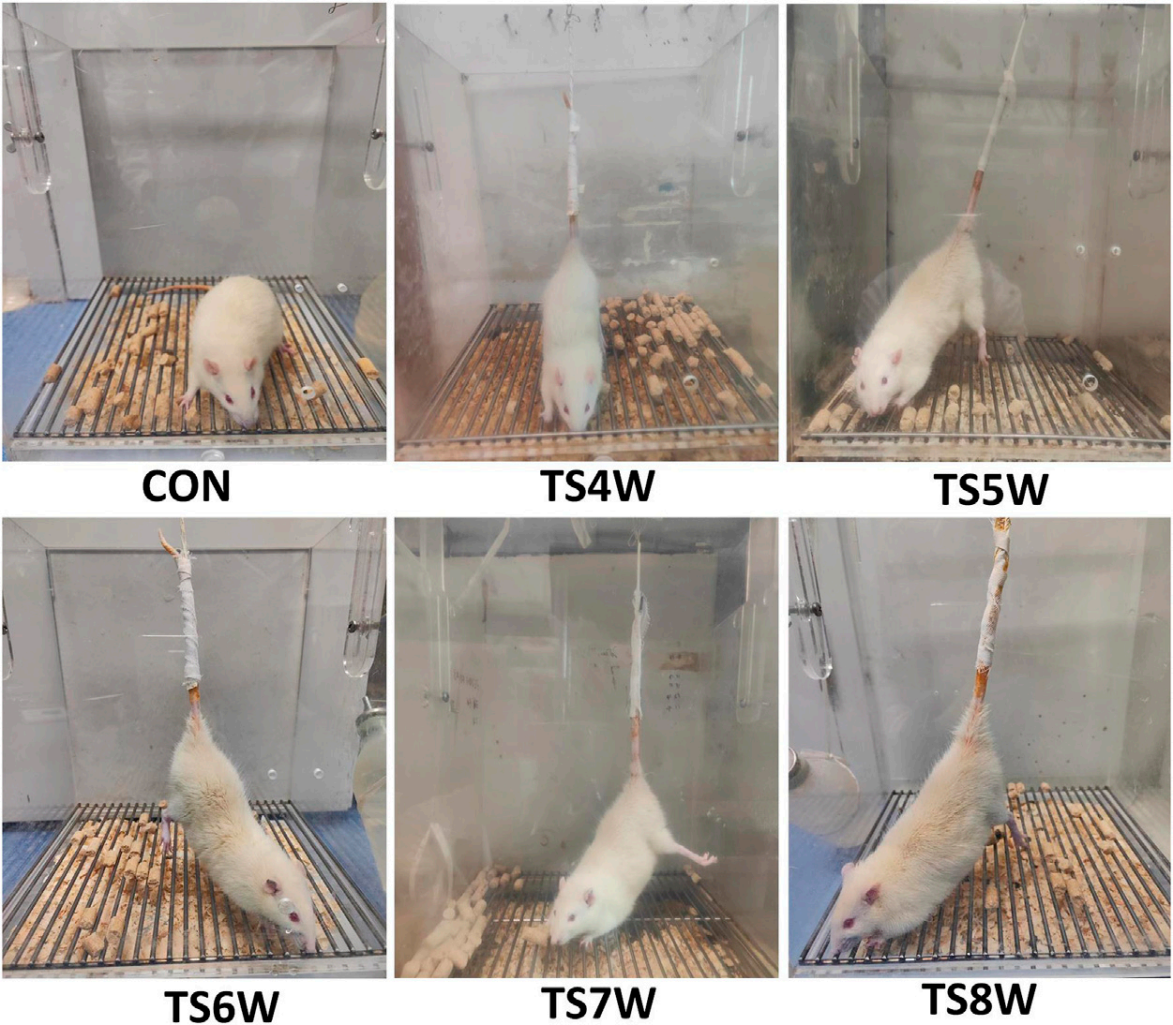
Rat tail suspension to simulate weightlessness.

Hind-limb unloading via tail suspension (TS group): According to the classic animal model of tail suspension, rats were placed in a 26 cm × 26 cm×30 cm Plexiglas box by tail suspension, maintaining the head at a -30° downward angle. First, the tail of each rat was cleaned and air-dried. The traction band was attached laterally along the proximal portion of the tail and held in place using three circular strips of tape. The traction band was then attached to a stainless-steel rod, allowing the rat to rotate freely at 360°. Indexes were measured at 4 and 8 weeks of tail suspension. Control without hind-limb unloading (CON group): Rats were reared in a single cage without tail suspension in the same environment. All rats in both groups were allowed to eat and drink freely (Figure 1).

### 2.3 Electroretinogram (ERG)

Before the test, the experimental animals were placed in a homemade dark adaptation box for >12 h. Subsequently, anesthesia was administered 3 mL/kg of 1% sodium pentobarbital (Sigma, St Louis, MO, United States) by intraperitoneal injection. After corneal surface anesthesia, 50 µL of 10% Librium (Jilin Shengda Animal Pharmaceutical Co., Ltd.), tropicamide (Shenyang Xingji Corporation), and orbucaine hydrochloride eye drops were administered. ERG was then recorded by full-field stimulation using a computer system (MonPack 3; Metrovision, France) in accordance with the procedure of the International Society for Clinical Electrophysiology of Vision (ISCEV). Electrodes (recording electrode Ag-Ag Cl corneal ring electrode, reference electrode stainless-steel buccal needle electrode, and ground electrode stainless-steel tail needle electrode) were placed, and the corresponding ERG indicators were recorded. The electrodes were removed after the recording was completed. Finally, gatifloxacin eye gel (Shenyang Xingji Corporation) was applied to the eyes.

### 2.4 OCTA of the fundus

Rats were anesthetized as described previously, and corresponding Optical coherence tomography angiography (OCTA) scans were performed using an OCTA Scan Head equipped with a rats’ objective lens. The right eye was dilated using 0.5% tropicamide eye drop. Gatifloxacin eye gel (Shenyang Xingji Corporation) was used to protect the cornea. OCTA images were obtained using 200,000 A-scans per second at a center wavelength of 1060 nm using a swept-source OCTA device (VG200S; SVision Imaging, Henan, China). The system was supplied with an eye-tracking utility based on an integrated confocal scanning laser ophthalmoscope to remove eye-motion artifacts. The axial and lateral tissue resolutions in the rat eye are 3.8 and 5 μm, respectively. A built-in wide-field 9 × 9 mm^2^ scanning protocol was applied. However, notably, the scanning area was regarding the human eye size (axial length: 24 mm), and the actual scanning area in the rat eye was about 3.9 × 2.4 mm^2^, assuming an axial length of 6.3 mm. The choroid in the SS-OCT images was defined as the area from the retinal pigment epithelium (RPE)–Bruch’s membrane complex to the choroid–sclera interface. After semiautomatic choroidal segmentation with a custom algorithm developed in MATLAB R2017a (MathWorks, Natick MA, United States), segmentations of RPE–Bruch’s membrane complex and choroid–sclera interface was adjusted manually by a trained examiner (PW) (Tan et al., 2020).

The choroidal and retinal thicknesses were manually measured using representative B-scans taken roughly 1.5 mm away from the ONH. The ONH’s position was confirmed using an enface view of the retina.

**FIGURE 2:**
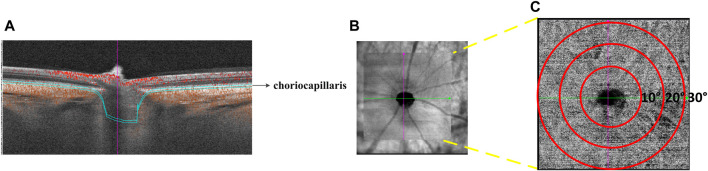
Illustration of choriocapillaris blood perfusion analysis. **(A)** Cross-sectional structure combined with flow OCT image with choriocapillaris segmentation lines. **(B)** OCTA scan region of 30° × 30°. **(C)** Magnified the face OCTA choriocapillaris image.

Choroidal vasculature beds: The choroid is a vascular bed that supplies nutrients and oxygen to the RPE and outer layers of the retina. It comprises larger vessels and a highly fenestrated capillary bed called the choriocapillaris. The choroidal images were obtained with a raster scan protocol of 512 horizontal B-scans that covered an area of 30° × 30° centered on the optic nerve. Each B-scan contained 512 A-scans and was repeated twice and averaged. The OCTA images were obtained by the SVision SS-OCTA algorithm. We analyzed en-face angiograms of the choriocapillaris slab, defined by a layer starting at the retinal pigmented epithelium (RPE) and ending around 8 µm below RPE. A maximum projection was applied on the segmented volumes to generate the en-face angiograms. Projection artifacts from retinal vessels were removed by the algorithm (Figure 2).

### 2.5 Pathological examination of retinal sections

Retinal histological alterations were visualized using HE staining of rat eye sections. The rats were euthanized with a lethal dose of sodium pentobarbital, and their eyeballs were rapidly removed and enucleated. The eye was placed in the prepared eyeball fixation solution (formaldehyde solution, acetic acid, 75% alcohol, Normal saline = 7:1:2:10), fixed for 24 h, dehydrated, and embedded to prepare wax blocks. Sagittal sections (4 µm) were then cut along the vertical direction of the optic nerve papilla using a paraffin microtome. Ten consecutive sections were prepared for the study. The slices were then dehydrated, stained with hematoxylin and eosin (HE), and observed under a light microscope (DP71; Olympus, Japan). Each optic disc-centered segment was inspected every 0.5 mm in the nasal and temporal orientations. Five points were taken. Due to the condensed cell arrangement in the outer nuclear layer (ONL), precise cell quantification was challenging. HE-stained sections were employed to evaluate the ONL by determining cell layers and measuring thickness. The identical number of sections and magnification was utilized for cell counting. Cell layers in the ONL of HE-stained sections were directly counted from the captured images.

### 2.6 Transmission electron microscopic observation of ultrastructural changes in the retina

Prefixed with a 3% glutaraldehyde, then the retinal tissue dehydrated in series acetone, infiltrated in Epox 812 for a longer, and embeded. The semithin sections were stained with methylene blue and Ultrathin sections were cut with diamond knife, stained with uranyl acetate and lead citrate. Sections were examined with JEM-1400-FLASH Transmission Electron Microscope. ImageJ software was deployed to observe cell morphology and structure. The surface area indicates the size of mitochondria in square micrometers (µm^2^).

### 2.7 TUNEL staining of retinal sections

Following the instruction in the TUNEL assay kit (Roche Diagnostics, United States), retinal sections were dewaxed and washed twice with xylene. The TUNEL reaction mixture was added after gradient dehydration with alcohol. The sections were then incubated in a wet box for 2 h. After washing with phosphate-buffered saline (PBS), the sections were restained with 4′,6-diamidino-2-phenylindole (DAPI). Fluorescein isothiocyanate (FITC) was added for retinal cell apoptosis detection by laser confocal microscopy (LSM 800, Zeiss, Germany). Nuclei show blue fluorescence, whereas apoptotic cells emit green fluorescence. Five fields were randomly selected from each tissue slice. Apoptosis rate = (number of apoptotic cells with green fluorescence/total number of cells) × 100.

### 2.8 Immunofluorescence staining of retinal sections

Paraffin-embedded sections were deparaffinized and dehydrated. Endogenous peroxidase activity was blocked with 3% H_2_O_2_ for 15 min, followed by three 5 min washes with PBS (0.1 mM, pH 7.2). Antigens were retrieved by boiling (100 C) in citric acid buffer (PH 6.0) for 20 min. Next, the sections were treated with 10% goat serum (including 0.3% Triton) for 1 h to block non-specific labeling. The sections were then incubated overnight at 4 °C with primary antibody against ZO-1 (Proteintech, 21773-1-AP, Wuhan, Hubei Province, China), Cx43 (Cell Signaling Technology, #3512, Danvers, MA) at 1:200 dilution. Slides incubated without the primary antibody served as controls. The slides were washed thrice with PBS and incubated for 1 h with IgG (H + L) and Cy3 fluorescence secondary antibody (EK022; Zhuangzhi, Xi’an, China) at 1:400 dilution. Nuclei were stained by incubating the sections in 100 ng/mL DAPI after rinsing thrice with PBS. A confocal microscope (LSM 800, Zeiss, Germany) was used for observation.

### 2.9 Western blotting analysis of Bax, Bcl-2, caspase-3, Cx43, ZO-1, and occludin expression

Protein was extracted from rat retina and choroid, and the protein concentration was determined using the BCA method. Briefly, 50 µL of 5x loading buffer was added to each protein tube, followed by boiling at 100°C for 15 min to denature the protein. Next, the denatured protein samples were subjected to SDS-RAGE gel electrophoresis and transferred to a PVDF membrane for 90 min. Occludin (Proteintech, 27260-1-AP, Wuhan, Hubei Province, China), Bcl-2 (Proteintech, 26593-1-AP, Wuhan, Hubei Province, China), and caspase-3 (Proteintech, 10380-1-AP, Wuhan, Hubei Province, China) at 1:1000 dilution. Cx43 (Cell Signaling Technology, #3512, Danvers, MA), Bax (Proteintech, 27155-1-AP, Wuhan, Hubei Province, China), LC3B (Abcam, ab192890, Cambridge, MA) at 1:2000 dilution, ZO-1 (Proteintech, 21773-1-AP, Wuhan, Hubei Province, China) at 1:4000 dilution, were added to the blocking solution, followed by incubation with shaking at room temperature for 1–3 h. After that, the samples were washed thrice with Tween-PBS for 10 min each. Next, the samples were incubated with the secondary antibody β-actin (Proteintech, Wuhan, Hubei Province, China) at room temperature with shaking for 1–2 h. Subsequently, the samples were washed thrice with Tween-PBS for 10 min each. Enhanced chemiluminescence detection reagents (Millipore, United States) were used to detect samples. The bands were obtained and analyzed using the FUSION FX SPECTRA (Vilber, France). The protein expression was compared with the β-actin expression as an internal reference to represent the relative protein expression level.

### 2.10 Statistical analysis

The results are presented as the mean ± SEM. Dunnett test was used to compare the experimental and control groups. Differences were considered significant at *p* < 0.05. Differences among groups were estimated by repeated variance analysis, Differences were considered significant at *p* < 0.05. Statistical analyses were performed using GraphPad Prism 8.0 (GraphPad Software, United States) and SPSS 23.0 (IBM Corporation, United States).

## 3 Results

### 3.1 Effects of simulated weightlessness in tail suspension on body weight and relative mass of soleus muscle in rats

**FIGURE 3:**
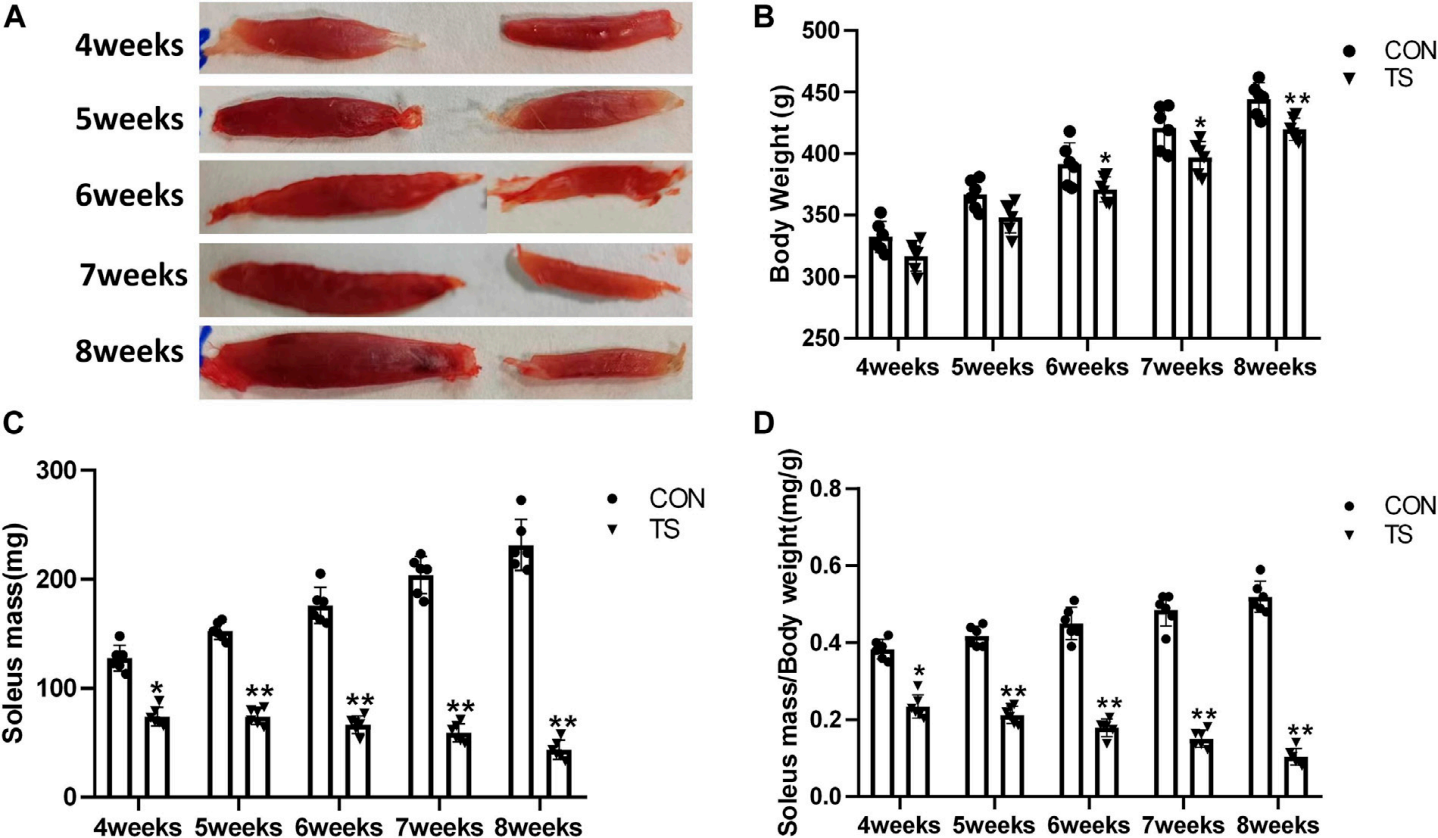
Body weight and relative mass of soleus muscle of rats in each group. **(A)** Soleus muscle images of rats in each group. **(B)** Weight of rats in each group. **(C)** Wet weight of soleus muscle in each group. **(D)** Relative mass of rat soleus muscle in each group. n = 6 rats per group. *p < 0.05, **p < 0.01, as compared with control group.

The TS group showed weight loss compared with the control group (Figure 3A). Specifically, a significant weight reduction was observed after 7 weeks of tail suspension (*p* < 0.05; Figure 3B). These findings indicated that tail suspension simulated weightlessness had a discernible impact on the growth of rats.

The ratio of soleus muscle mass to body mass serves as the relative mass of soleus muscle. Compared with the control group, The wet weight and relative mass of soleus muscles in the TS group rats decreased as the tail suspension duration increased. A significant decrease was observed after 4 weeks of simulated weightlessness (*p* < 0.05; Figures 3C, D), confirming the suitability of tail suspension as a method to simulate microgravity.

### 3.2 Choroidal thickness and choroidal capillary blood flow in each group

**FIGURE 4:**
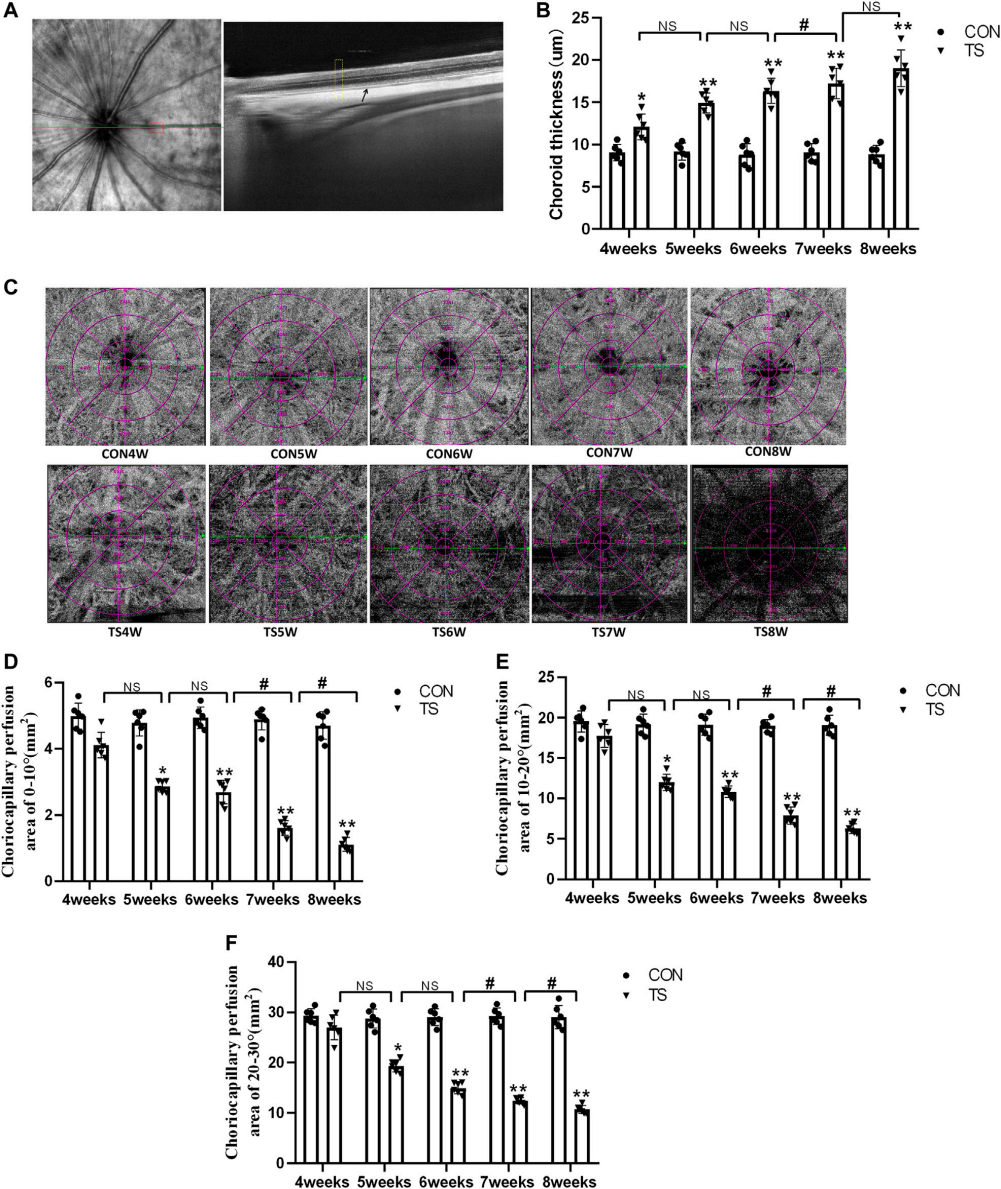
Choroidal thickness and choroidal capillary blood flow chart of rats in each group. **(A)** Typical OCTA image, the yellow box shows the area of the choroidal structure analysis, and the black arrow displays the choroidal structure. **(B)** Choroidal thickness in the OCTA image. *p < 0.05, **p < 0.01, as compared with control group; ^#^ *p* < 0.05: the TS7W group compared with the TS6W group. **(C)** Illustration of choriocapillaris blood perfusion analysis. **(D–F)** Choroidal capillary blood flow analysis image. *p < 0.05, **p < 0.01, as compared with control group; ^#^ *p* < 0.05: the TS7W group compared with the TS6W group and the TS8W group compared with the TS7W group. n = 6 rats per group.

The choroidal thickness of rats in each group was measured using OCTA (Figure 4A). As shown in Figure 4B, compared with the CON group, choroidal thickness in the TS group increased with the extension of the tail suspension time, significantly increasing after 4 weeks (*p* < 0.05). Additionally, Compared with the TS6W group, choroidal thickness in the TS7W group increased significantly (*p* < 0.05).

In the OCTA images, the choroidal capillary layer is characterized by uniformly distributed black and white punctate, snowflakes, and granules. As shown in Figure 4C, choroidal capillaries in the CON group were black and white punctate with uniform distribution and adequate blood perfusion. In contrast, the TS group exhibited decreased blood perfusion area of choroidal capillaries and disordered and uneven choroidal capillary distribution. Each group’s perfusion area of choroidal capillaries was analyzed based on the ETDRS ring (multiple concentric ring grids) centered on the papilla. As shown in Figures 4D–F, at a ring distance of 0°–10°, 10°–20°, and 20°–30° from the papilla, the perfusion area of choroidal capillaries was less in the TS group than in the CON group. The area of choroidal capillary perfusion was significantly reduced after 5 weeks of caudal suspension (*p* < 0.05). Furthermore, the perfusion area of choroidal capillaries in the TS group decreased as the tail suspension duration increased. Specifically, the perfusion area of choroidal capillaries was significantly reduced in the TS7W group compared with the TS6W group (*p* < 0.05). These data indicated that tail suspension in simulated weightlessness significantly impacted the choroidal circulation after 7 weeks, resulting in choroidal congestion and thickening, choroidal blood circulation disruption, and decreased choroidal microcirculation perfusion area.

### 3.3 Effects of simulated weightlessness on the ultrastructure of choroidal vascular endothelial cells in rats

**FIGURE 5:**
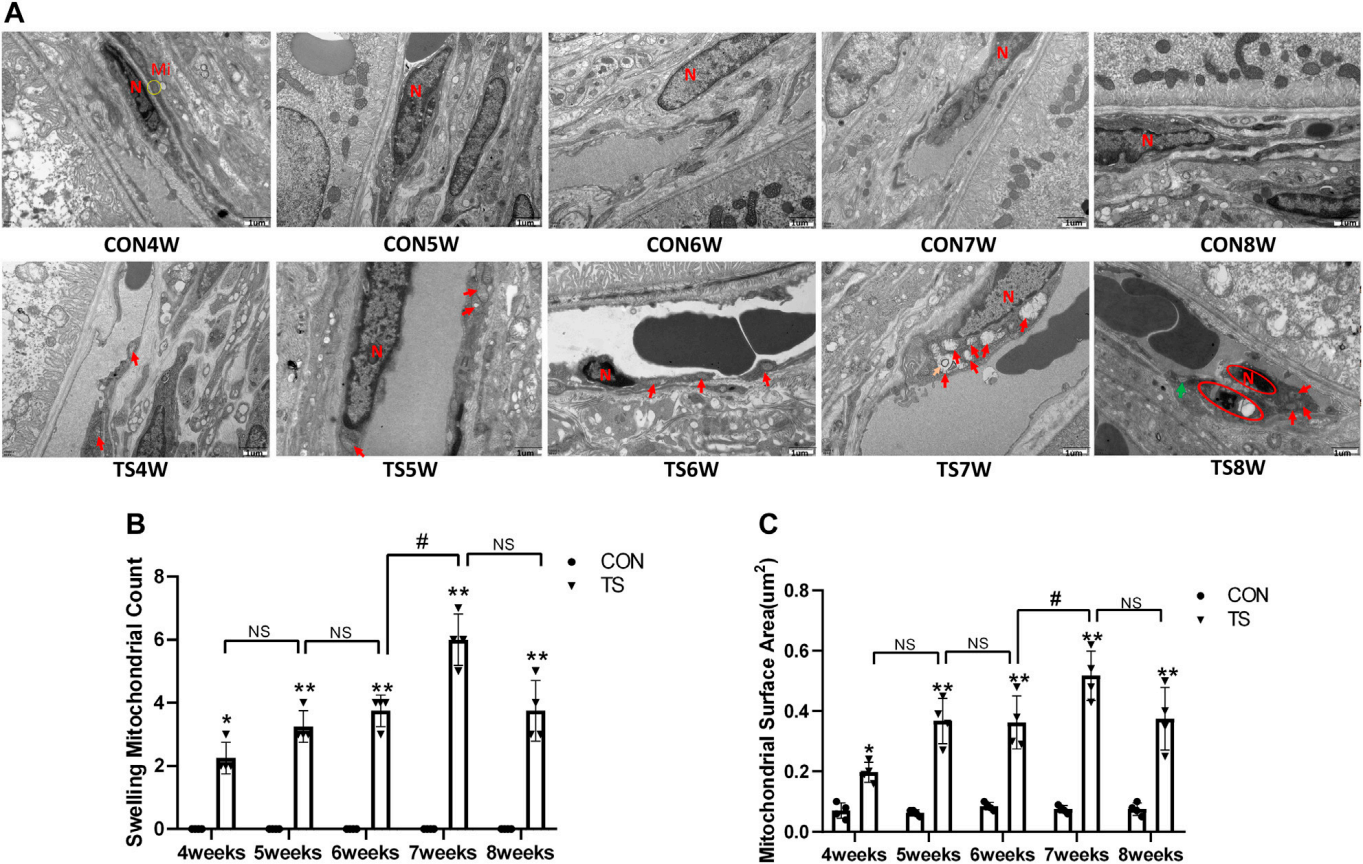
Ultrastructure of choroidal vascular endothelial cells in rats. Mi represents mitochondria. **(A)** The ultrastructure of choroidal vascular endothelial cells observed by transmission electron microscopy. **(B)** Counting the swollen mitochondria in each cell. **(C)** Mean surface area of mitochondria on transmission electron microscopy images. The yellow circle represents the mitochondrial structure. Red arrows indicate swollen mitochondrial structure. Orange arrows symbolize mitochondrial cristae myelinating degeneration. Green arrows symbolize autophagy. Red circles mark vascular endothelial cells apoptosis. N stands for nucleus. Scale bar = 1 μm n = 4 rats per group. *p < 0.05, **p < 0.01, as compared with control group; ^#^ *p* < 0.05: the TS7W group compared with the TS6W group.

The ultrastructure of choroidal vascular endothelial cells was observed by electron microscopy. As shown in Figure 5A, the results showed that blood vessels in the CON group were well dilated; the nuclei morphology of vascular endothelial cells were intact; and the structure of choroidal capillary endothelial cells was intact. Compared to the CON group, the TS group’s blood vessels were well-dilated by 4 weeks, but most mitochondria in the vascular endothelial cells were subtly swollen. By the fifth week, the TS group’s blood vessels were well-dilated, although the vascular endothelial cells were somewhat swollen. By the sixth week, red blood cells were observed in the TS group’s blood vessels. Moreover, the mitochondria of vascular endothelial cells showed obvious swelling; erythrocyte aggregation was observed in the blood vessels. In the 7-week tail suspension group, erythrocyte aggregation was found in the blood vessels, certain vascular endothelial cells were susceptible to apoptosis (nuclear pyknosis, chromatin aggregation, and cytosolic electron density increase), and most mitochondria in the cytoplasm are swollen and vacuolate in appearance, while a small number of mitochondrial cristae undergo myelin-like transformation. By the eighth week, erythrocyte aggregation, mitochondrial swelling, vascular endothelial cell apoptosis, and autophagy were observed in the TS group’s blood vessels.

Compared with the control group, with the extension of tail suspension time, TS group exhibited a significant increase in the number of swollen mitochondria and the mitochondrial area (*p* < 0.05, Figures 5B, C). Notably, the number and area of mitochondrial swelling significantly increased in the TS7W group compared with the TS6W group (*p* < 0.05). These findings indicated that tail suspension induced mitochondrial damage and functional changes in choroidal capillary endothelial cells. The extent of mitochondrial damage was more significant after 7 weeks of tail suspension, consistent with the time point of significant impairment in choroidal microcirculation.

### 3.4 Effects of simulated weightlessness on the ultructure of rat retinal pigment epithelial cells

**FIGURE 6:**
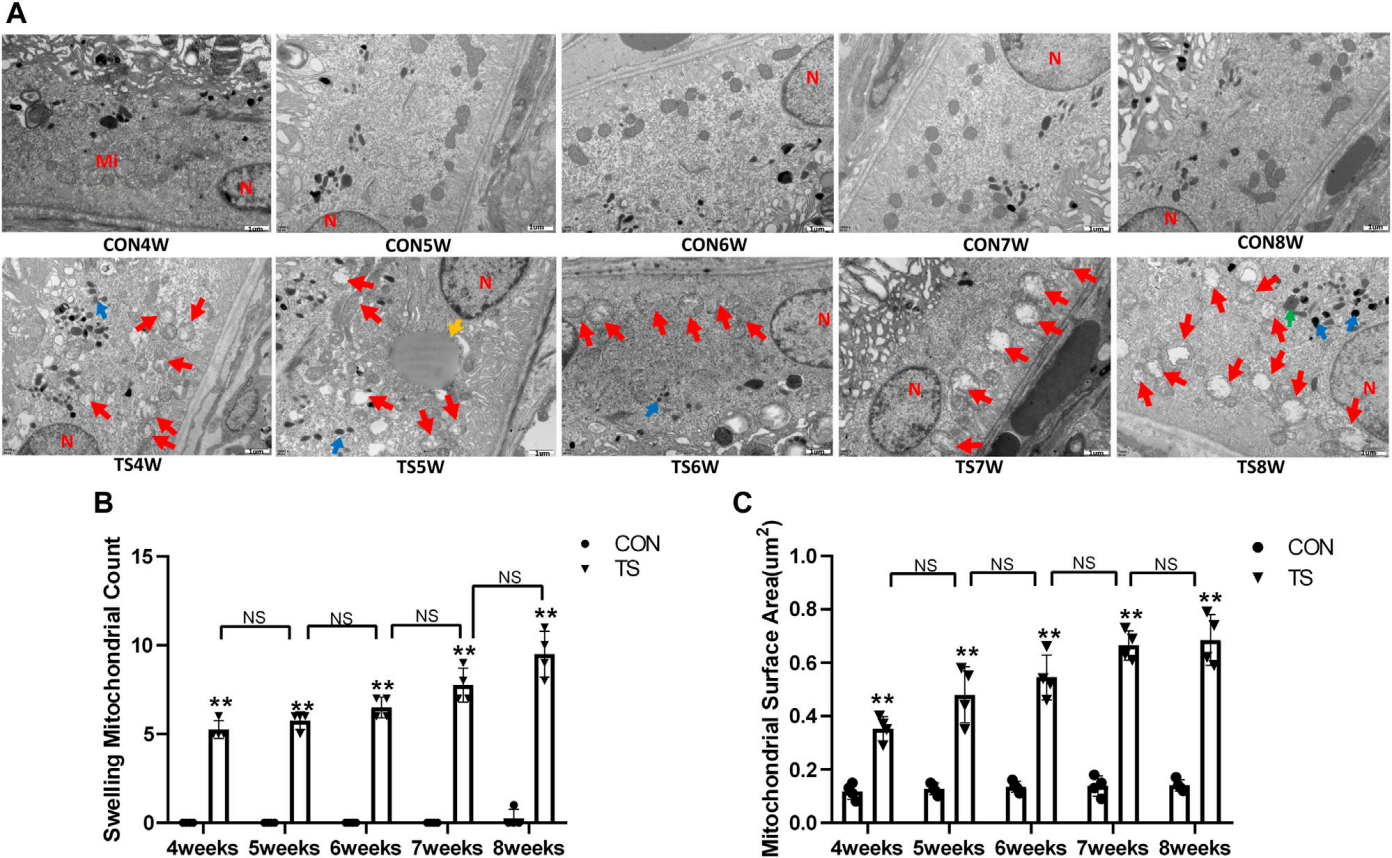
The ultrastructure of retinal pigment epithelial cells in rats subjected to simulated weightlessness. **(A)** The ultrastructure of retinal pigment epithelial cells observed by transmission electron microscopy. **(B)** Counting the swollen mitochondria in each cell. **(C)** Mean surface area of mitochondria on transmission electron microscopy images. Mi represents mitochondria. The yellow circle represents the mitochondrial structure. Red arrows indicate swollen mitochondrial structure. Blue arrows indicate pigment granule structure. Green arrows represent autophagy structure. Yellow arrows represent fat droplets. The orange circle represents the rough endoplasmic reticulum. N indicates nucleus. Scale bar = 1 μm n = 4 rats per group. *p < 0.05, **p < 0.01, as compared with control group.

The ultrastructure of the retinal pigment epithelial cells was observed under an electron microscope. As shown in Figure 6A, the results showed that the morphology and structure of pigment epithelial cells in the CON group were normal; the mitochondria and other organelles were intact and clear; the pigment cells’ nuclei were round; the chromatin was evenly distributed; and the nuclear membrane was visible and complete. Organelles, like mitochondria, rough endoplasmic reticulum, and ribosomes, were observed in the cytosol, and their structures were intact and clear. By the fourth week, most mitochondria in the pigment epithelial cells of rats in the TS group were slightly enlarged, and there were more pigment granules than in the CON group. By the fifth week, several mitochondria in the cytoplasm of pigmented epithelial cells showed mild swelling (crista fracture and dissolution). Small fat droplets were also seen in the TS group. By the sixth week, the mitochondria in the cytoplasm of pigment epithelial cells were swollen (critical fracture and dissolution). Most of the mitochondria were swollen in the TS group by the seventh week. By the eighth week, most mitochondria were swollen, autophagy was observed sporadically, and pigment granules were abundant in the pigment epithelial cells of the TS group.

Compared with the control group, with extended tail suspension time, the number of swollen mitochondria in TS group significantly augmented, as was the mitochondrial area (*p* < 0.05, Figures 6B, C). Such findings suggest that tail suspension can cause mitochondrial damage and functional changes in retinal pigment epithelial cells.

### 3.5 Expression of Cx43, ZO-1, and occludin in each group

**FIGURE 7:**
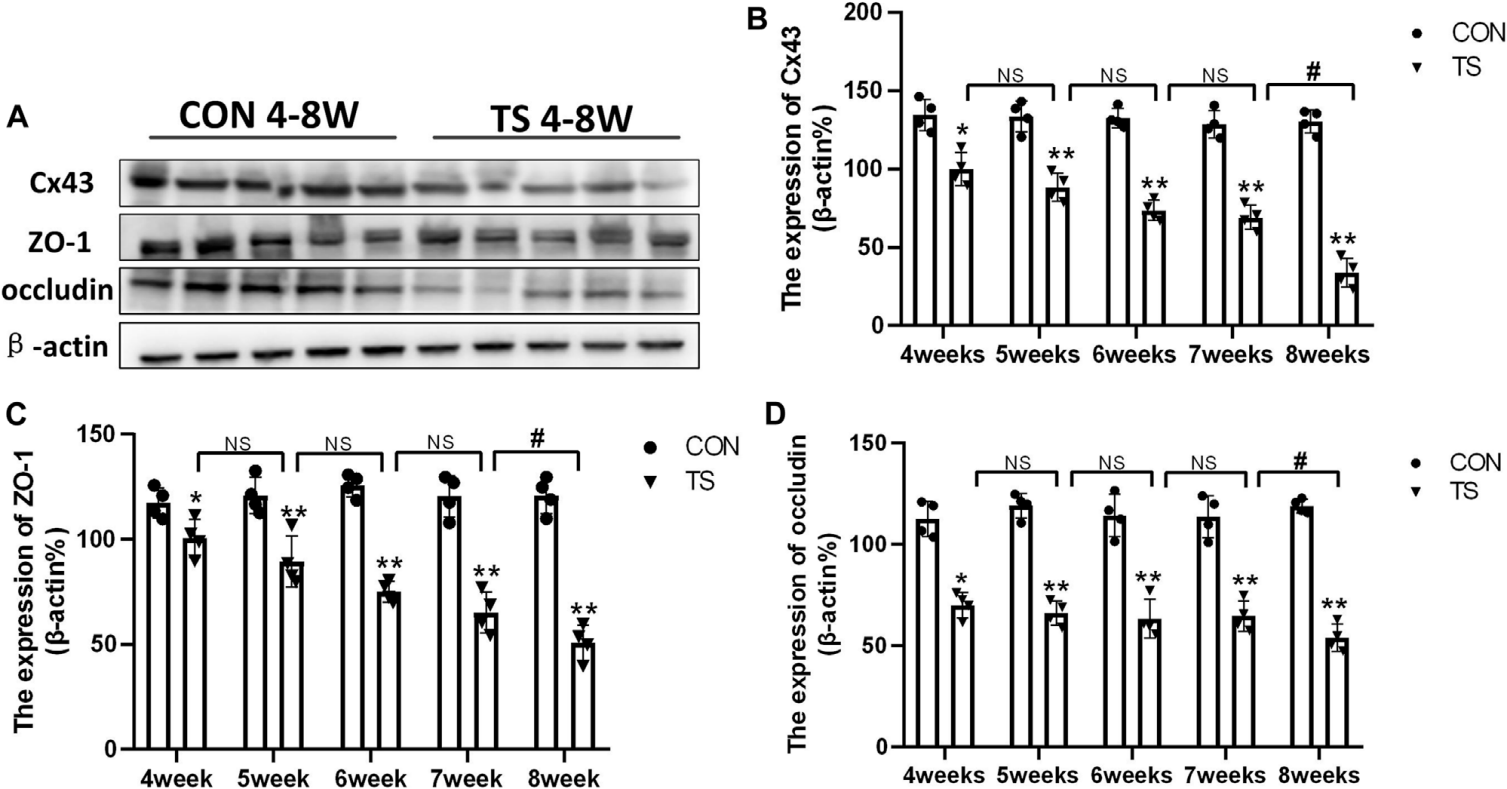
Expression of Cx43, ZO-1, and occludin in the retina and choroidl of rats in each group. **(A)** The protein expression levels of Cx43, ZO-1, and occludin were evaluated by western blot in the retina and choroidl. **(B–D)** Quantitative analysis of Cx43, ZO-1, and occludin in the retina and choroidal of rats in each group. n = 4 rats per group. *p < 0.05, **p < 0.01, as compared with control group; ^#^ *p* < 0.05: the TS8W group compared with the TS7W group.

Cx43, ZO-1, and occludin, as gap junction molecules, are significant in maintaining the junction and integrity of endothelial cells. As shown in Figures 7A–D, the results showed that the expression of Cx43, ZO-1, and occludin decreased with the extension of tail suspension time, and Cx43, ZO-1, and occluding expression decreased significantly after 4 weeks of tail suspension (*p* < 0.01). Additionally, we observed a significant decrease in the expression of Cx43, ZO-1, and occlusion in the TS8W group compared with the TS7W group (*p* < 0.05). The results indicated that tail suspension simulated weightlessness could disrupt the outer blood-retinal barrier in rats, reducing expressions of the gap junction proteins Cx43, ZO-1, and occluding. Notably, the damage to the outer blood-retinal barrier was more significant after 8 weeks of tail suspension.

### 3.6 Indicating localization of ZO-1 and Cx43 by immunofluorescence

**FIGURE 8:**
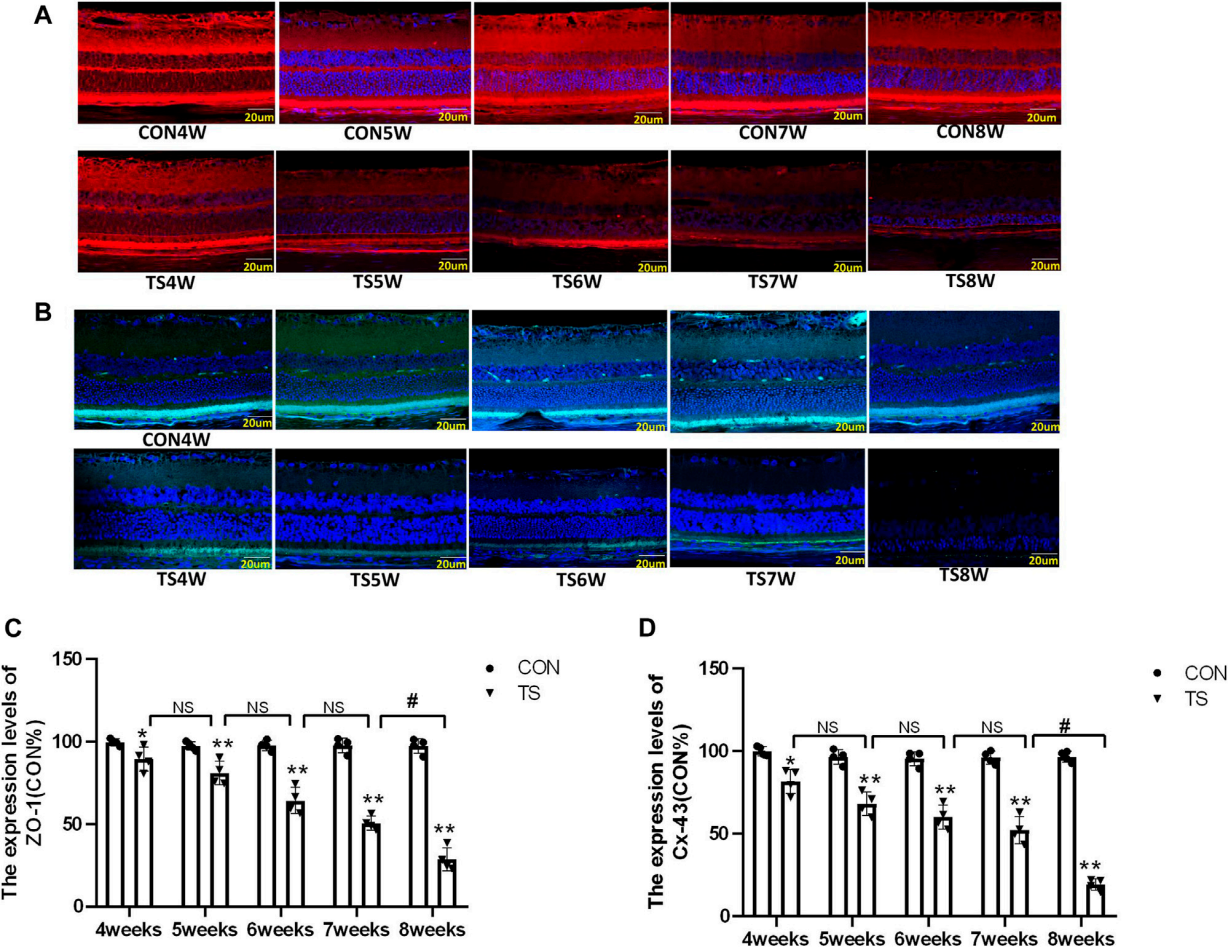
The expression of ZO-1 and Cx-43 in the outer blood-retinal barrier of each group. **(A)**, **(C)** Represented the expression of ZO-1 and Cx-43 in the blood-retinal barrier of rats belonging to distinct groups, Scale bar = 20 μm. **(B)**, **(D)** The relative quantitative analysis of the ZO-1 and Cx-43 in the outer blood-retinal barrier in each group. n = 4 rats per group. *p < 0.05, **p < 0.01, as compared with control group; ^#^ *p* < 0.05: the TS8W group compared with the TS7W group.

Cx-43 and ZO-1 expressions in the outer blood-retinal barrier were observed by immunofluorescence labeling. We observed that the Cx-43 and ZO-1 expressions in the external blood-retinal barrier of rats in the tail suspension group decreased with the extension of tail suspension time (Figures 8A, B). Three visual fields were randomly picked from the nasal and temporal sides to calculate the fluorescence expression intensity, with the papilla in the center. The results were as follows (Figures 8C, D): The expressions of Cx-43 and ZO-1 in the external blood-retinal barrier of rats in the tail suspension group were significantly lower than those in the control group after 4 weeks of tail suspension (*p* < 0.05). Moreover, Cx43, ZO-1, and occlusion expression significantly reduced in the TS8W group compared with the TS7W group (*p* < 0.05). Immunofluorescence staining was consistent with western blotting results.

### 3.7 Effects of simulated weightlessness on retinal function in rats

**FIGURE 9:**
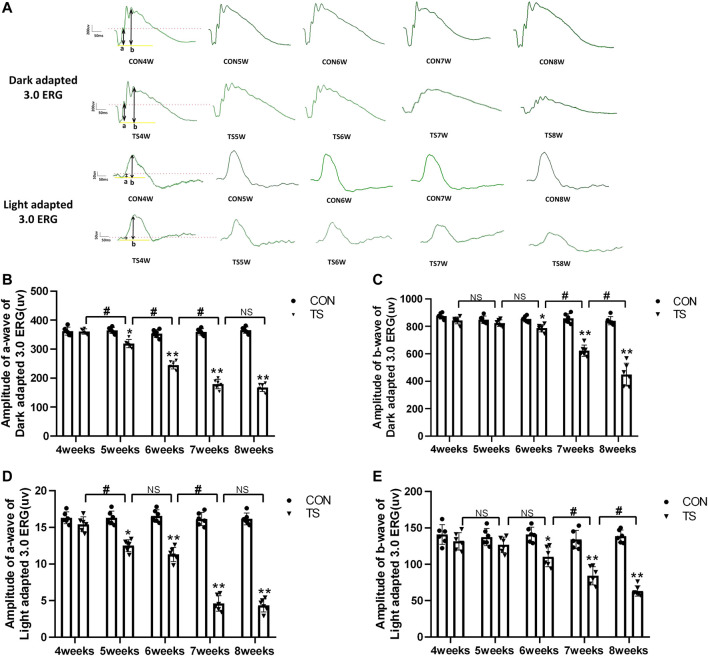
Retinal function in rats subjected to simulated weightlessness as assessed by electroretinogram (ERG). **(A)** Typical ERG waveforms of the control and tail suspension groups. **(B)** Amplitudes of the a-waves of the dark-adapted 3.0 ERG. *p < 0.05, **p < 0.01, as compared with control group; ^#^ *p* < 0.05: the TS5W group compared with the TS4W group, the TS6W group compared with the TS5W group and the TS7W group compared with the TS6W group. **(C)** Amplitudes of the b-waves of the dark-adapted 3.0 ERG.*p < 0.05, **p < 0.01, as compared with control group; ^#^ *p* < 0.05: the TS7W group compared with the TS6W group and the TS8W group compared with the TS7W group; **(D)** Amplitudes of the a-waves of the light-adapted 3.0 ERG response.*p < 0.05, **p < 0.01, as compared with control group; ^#^ *p* < 0.05: the TS5W group compared with the TS4W group and the TS7W group compared with the TS6W group; **(E)** Amplitudes of the b-waves of the light-adapted 3.0 ERG response.*p < 0.05, **p < 0.01, as compared with control group; ^#^ *p* < 0.05: the TS7W group compared with the TS6W group and the TS8W group compared with the TS7W group. n = 6 rats per group.

ERG is an important tool for evaluating the retina’s overall function. To clarify the influence of microgravity on the retina, the overall function of the retina in rats exposed to simulated microgravity by tail suspension was evaluated using ERG. The results showed that retinal function decreased significantly after tail suspension. Light adaptation 3.0 and dark adaptation 3.0 response amplitudes were less in the TS group than in the CON group (Figure 9A). The dark-adapted 3.0 ERG a-wave mainly reflects rod and cone functions. Compared to the CON group, the a-wave amplitude of the TS group showed a decreasing trend after 4 weeks and was significantly decreased by the fifth week (*p* < 0.05, Figure 9B). The dark-adapted 3.0 ERG b-wave amplitude mainly reflects the combined response capacity of the rod, cone, and bipolar cells. Compared to the CON group, the b-wave amplitude of the TS group showed a decreasing trend after 4 weeks and was significantly decreased at 6 weeks (*p* < 0.05, Figure 9C). The amplitude in the light-adapted 3.0 ERG represents cone cell function. Cone cells and cone line-off bipolar cells contribute to the a-wave amplitude of the light-adapted 3.0 ERG. Compared to the CON group, the TS group showed a decrease in a-wave amplitude by 4 weeks and a significant decrease by 5 weeks (*p* < 0.05, Figure 9D). The b-wave amplitude in the light-adapted 3.0 ERG originates from bipolar cells of the On and Off cone systems. Compared to the CON group, the TS group showed decreased b-wave amplitude in the light-adapted 3.0 ERG by 4 weeks and a significant decrease after 7 weeks (*p* < 0.01, Figure 9E). Additionally, we observed a significant decrease in a-wave amplitudes of light adaptation 3.0 and dark adaptation 3.0 in the TS5W group compared with the TS4W group (*p* < 0.05, Figures 9B, D). Similarly, the b-wave amplitudes of light adaptation 3.0 and dark adaptation 3.0 significantly decreased in the TS8W group compared with the TS7W group (*p* < 0.05, Figures 9C, E). These findings showed that tail suspension simulated weightlessness significantly impacted retinal function after 7 weeks, consistent with the time point of significant disturbance in choroidal microcirculation.

### 3.8 Effects of simulated weightlessness on retinal morphology in rats

**FIGURE 10:**
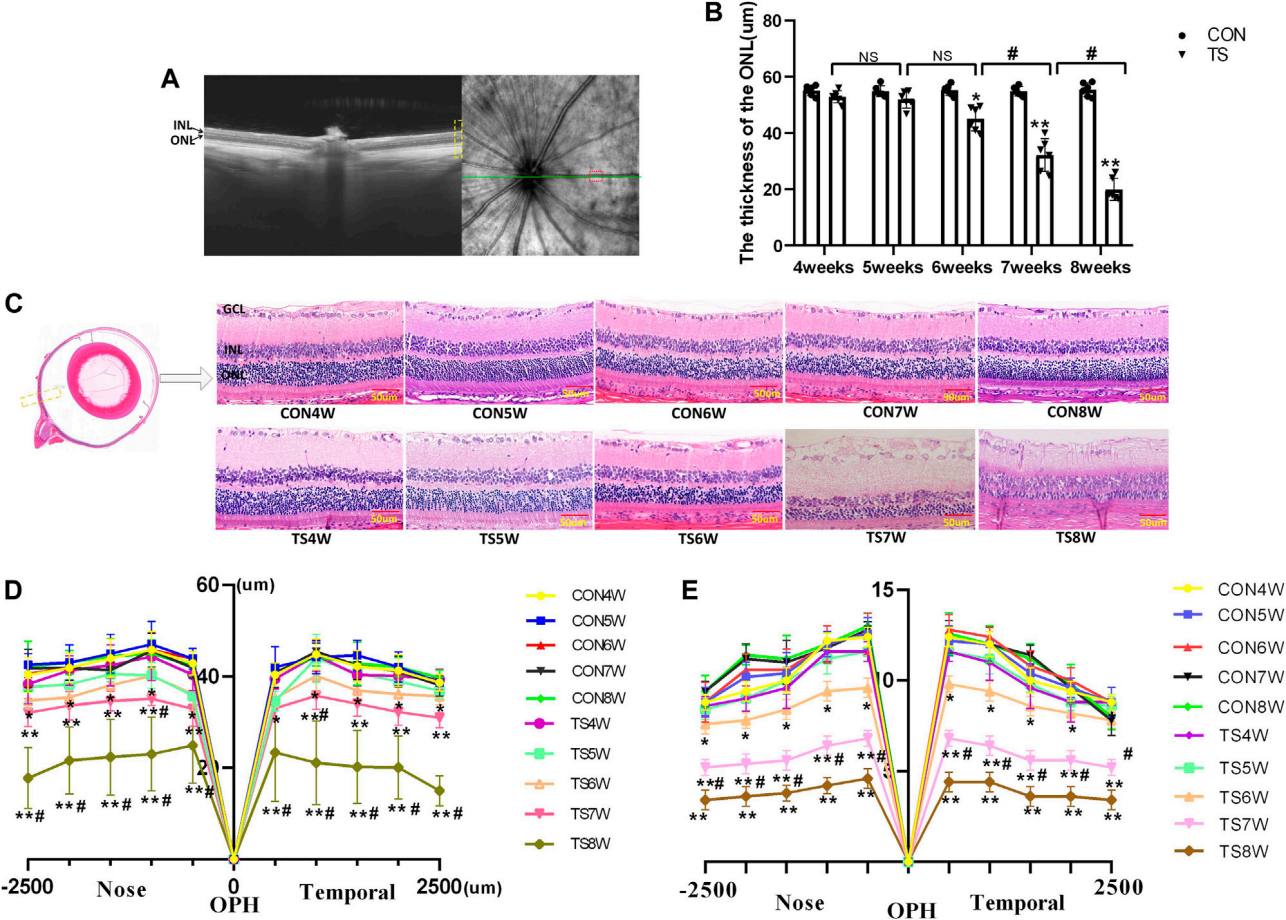
Retinal morphological structure in rats subjected to simulated weightlessness. **(A)** Typical OCTA labeled image. **(B)** OCTA image showing the retinal ONL thickness. **(C)** stained images of rat retinal sections from all groups and plots of rows of the ONL. Yellow boxes display areas where retinal structures are observed. **(D)** statistical maps of ONL thickness in different regions of the ONL of rats in each group. **(E)** statistical maps of ONL layer in different regions of ONL of rats in each group. ONL, outer nuclear layer; INL, inner nuclear layer; GCL, ganglion cell layer. Scale bar = 50 μm n = 6 rats per group. *p < 0.05, **p < 0.01, as compared with control group; ^#^ *p* < 0.05: the TS7W group compared with the TS6W group and the TS8W group compared with the TS7W group.

*In vivo* and *in vitro* evaluations of retinal morphology using OCTA and HE staining were performed on retinal slices to clarify the microgravity influence on retinal morphology. OCTA can be used to determine each layer of the retina based on different luminance reflection bands (Figure 10A). The OCTA analysis showed that the ONL thickness of the TS group decreased as the tail suspension duration increased. Specifically, a significant decrease in the ONL thickness of the TS group was observed after 6 weeks of tail suspension (*p* < 0.05, Figure 10B). Furthermore, a significant decrease in ONL thickness was observed in the TS8W group compared with the TS7W group (*p* < 0.05).

HE staining of the CON group showed clear tissue structure of the retina and normal morphological structure; the inner nuclear layer and outer nuclear layer cells were arranged regularly, and the boundary between the nucleus and cytoplasm was clear. By the fourth week of tail suspension, the retinal structure remained clear, with regular shape and neat structure edges, but the ONL cell structure was slightly loose, and the thickness and layer of ONL remained unchanged compared with that in the CON group. After 5 weeks of tail suspension, the distribution of retinal tissue structure became slightly irregular, and ONL cells became dispersed. ONL thickness and layer decreased as the tail suspension extended. By the sixth week, ONL thickness and cells decreased significantly (*p* < 0.05, Figures 10C–E). Moreover, the ONL thickness and cells of the retina in the TS group decreased as the tail suspension duration increased. We observed a significant decrease in the ONL thickness and cells ONL of the retina in the TS7W group compared with the TS6W group (*p* < 0.05). These data indicated that a 7-week period of tail suspension induced significant degenerative changes in the ONL, coinciding with the point at which tail suspension impairs the choroidal microcirculation. This observation supports the vital role of the outer blood-retinal barrier in providing nutritional support to the ONL. The trend of changes in histomorphology was consistent with the retina’s function.

### 3.9 Effects of simulated weightlessness on the ultrastructure of the retinal optic cell layer in rats

**FIGURE 11:**
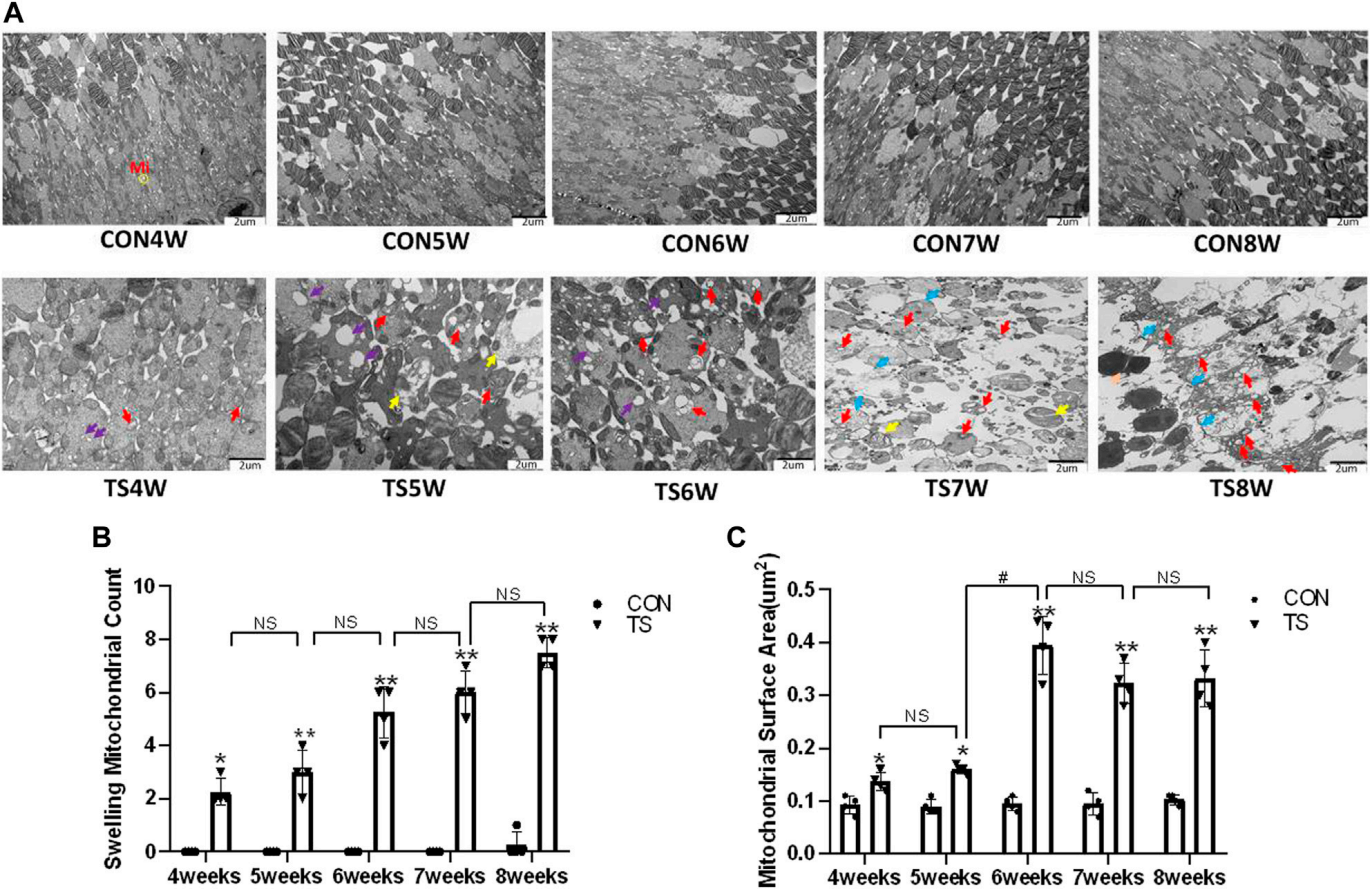
The ultrastructure of the retinal optic cell layer in rats subjected to simulated weightlessness. **(A)** The ultrastructure of retinal optic cell layer observed by transmission electron microscopy; **(B)** counting the swollen mitochondria in each cell; **(C)** mean surface area of mitochondria on transmission electron microscopy images.

Electron microscopy was used to investigate the ultrastructure of the membrane disc and inner segment mitochondria of retinal photoreceptor cells in each group. As shown in Figure 11A, the results showed that the mitochondrial structure in the inner segments of photoreceptor cells of rats in the CON group was clear and normal. A clear, intact, tight junction structure was observed between each cell. The structure was clear and organized. After 4 weeks, small vacuoles were observed in the inner segments of rod cells in the TS group, with normal morphological structure of mitochondria and clear and neatly arranged structure of the optic cell layer. By the fifth week, the inner segments of rod cells were lysed; the membrane disc structure in most areas was also solubilized; the morphological structure of the mitochondria was normal; many vacuoles were observed. Moreover, the structure of the optic cell layer was clear and arranged neatly in the TS group. By the sixth week, obvious mitochondria swelling of the inner segments of rod cells and more vacuoles were observed in the TS group. By the seventh week, the membrane disc structure was almost completely dissolved with only a few remains; a few mitochondria have mild swelling, and the cell membrane is discontinuous in some areas. By the eighth week, most of the membrane disc structures were dissolved; most of the mitochondria in the inner segments of rod cells were swollen. Moreover, the crista was disrupted, dissolved, or even disappeared; there were residual connection structures between rod cells and ONL cells; the space between ONL cells was significantly widened, and some inner segments of optic cells also exhibited cytoplasmic concentration and increased electron density.

Compared with the control group, as tail suspension time extended, the number of swollen mitochondria in TS group exhibited a significant increase, as was the mitochondrial area (*p* < 0.05, Figures 11B, C). Moreover, the area of mitochondrial swelling significantly increased in the TS6W group compared with the TS5W group (*p* < 0.05, Figure 11C). The results imply that tail suspension can cause mitochondrial damage and functional changes in retinal optic cell layer.

Mi represents mitochondria. The yellow circle represents the mitochondrial structure. Purple arrows indicate vacuoles. Red arrows indicate swollen mitochondria. Yellow arrows indicate dissolved membrane discs. Blue arrows indicate cell membrane discontinuity. Scale bar = 2 μm n = 4 rats per group. *p < 0.05, **p < 0.01, as compared with control group; ^#^ *p* < 0.05: the TS6W group compared with the TS5W group.

### 3.10 Apoptosis in retinal sections detected by TUNEL staining

**FIGURE 12:**
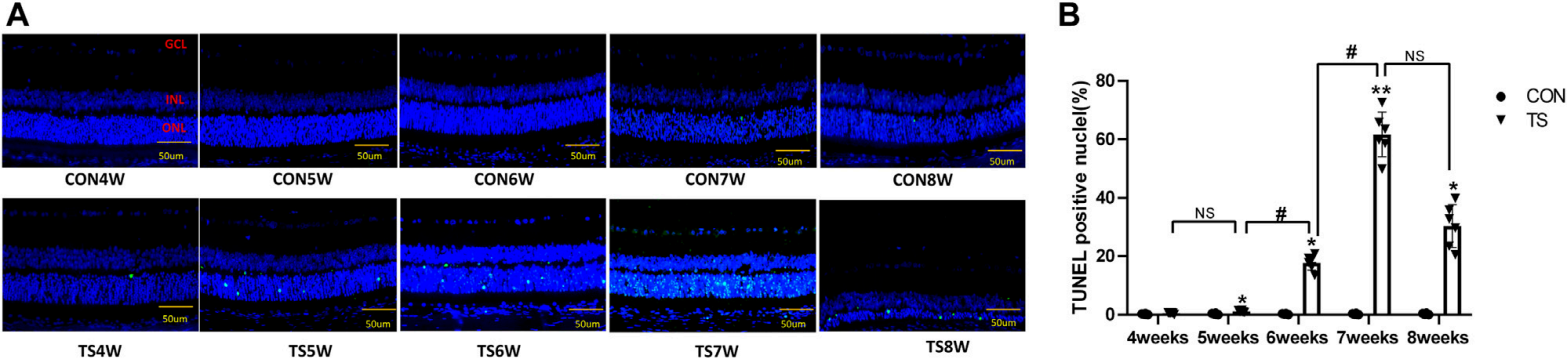
Terminal deoxynucleotidyl transferase deoxyuridine dUTP nick-end labeling (TUNEL) staining of rat retina in each group. **(A)** Retinal cell apoptosis in control (CON) and tail suspension groups was analyzed using TUNEL assay (×400 magnification). Blue and green stains indicate cell nuclei and apoptotic cells, respectively. **(B)** Positive apoptosis rates in rats’ outer retinal nuclear layer in each group. n = 6 rats per group. Scale bar = 50 μm *p < 0.05, **p < 0.01, as compared with control group; ^#^ *p* < 0.05: the TS6W group compared with the TS5W group and the TS7W group compared with the TS6W group.

Retinal apoptosis was examined by TUNEL assay in each group (Figure 12A). No apoptotic cells were observed in the retina of the CON group. The ONL of rats in the TS group showed different degrees of apoptosis. Compared to the CON group, the number of TUNEL-positive cells in the retinal tissue in the TS group increased with the extension of tail suspension time. The number of TUNEL-positive cells in the retinal tissue of rats increased significantly after fifth weeks of tail suspension (*p* < 0.05, Figure 12B). The apoptosis index of the ONL significantly increased in the TS6W group compared with the TS5W group (*p* < 0.05). Furthermore, the highest level of apoptosis in retinal tissue was observed after 7 weeks of tail suspension. The results indicated that the ONL cell count declined due to retinal damage-induced apoptosis.

### 3.11 Expression of caspase-3, Bax, Bcl-2 and LC3B in each group

**FIGURE 13:**
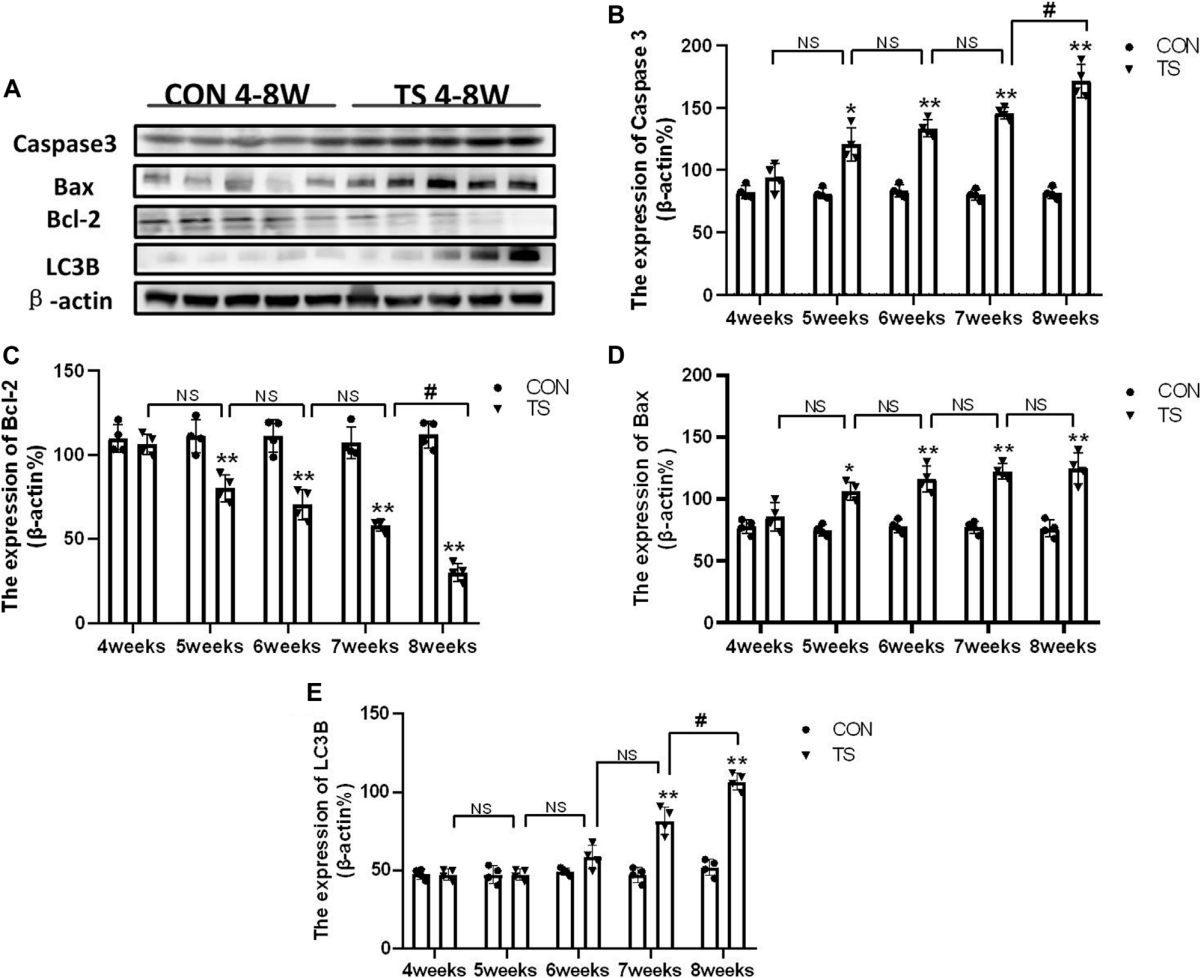
Expression of caspase-3, Bax, Bcl-2 and LC3B proteins in rat retina and choroidal in each group. **(A)** The protein expression levels of caspase-3, Bax, Bcl-2 and LC3B evaluated by western blot in the retina and choroidal. **(B–E)** quantitative analysis of Bax, Bcl-2, caspase-3, and LC3B in rat retina and choroidal in each group. n = 4 rats per group. *p < 0.05, **p < 0.01, as compared with control group; ^#^ *p* < 0.05: the TS8W group compared with the TS7W group.

Western blotting was employed to detect the expressions of apoptosis-related protein molecules such as caspase-3, Bax, and Bcl-2, as well as the autophagia-related protein molecules such as LC3B in the retinal and choroidal tissues of each group (Figure 13A). The expression of caspase-3, Bax, and LC3B increased, while Bcl-2 expression decreased in the TS group with the duration of tail suspension compared with the control group (*p* < 0.05, Figures 13B–E). Moreover, a significant increase in caspase-3 and LC3B expression was observed in the TS8W group compared with the TS7W group (*p* < 0.05, Figures 13B, E), while Bcl-2 expression significantly decreased in the TS8W group (*p* < 0.05, Figure 13C). Therefore, the western blotting results confirmed that the decrease in ONL was caused by the apoptosis of ONL cells, as confirmed by the TUNEL assay results. In addition, autophagy participates in the pathological process of tail-suspended simulated weightlessness retinal degeneration.

## 4 Discussion

In this study, a rat model was used to examine the temporal patterns of choroid circulation disorders and retinal degeneration under simulated weightlessness conditions. The effect of weightlessness on microcirculation has attracted more attention (Bimpong-Buta et al., 2018; Bimpong-Buta et al., 2020). Microcirculation is vital for blood flow regulation and tissue and organ function maintenance. The evaluation of microcirculation has become increasingly important in clinical practice, particularly for severe patients. Microcirculation has been identified as one of the most important predictors of mortality, and prompt examination of microcirculation has been regarded as one of the methods for enhancing treatment techniques (Jung et al., 2016). The choroidal microcirculation is rich in blood flow and is the vascular tissue with the highest blood flow in the whole body. It plays a vital role in maintaining the homeostasis of the retinal environment. OCTA signals appeared sensitive to microcirculation change (Wu et al., 2021). In this study, choroidal thickness and density of choroidal capillary in each group were dynamically observed by OCTA *in vivo*. The results showed that the choroidal thickness of each experimental group increased with an increase in tail suspension time, which was consistent with the previous finding (Figure 4B). However, a recent study reported that the thickness of the choroidal of 10-week-old mice was thinner when they returned to earth after 35 days in space (Overbey et al., 2019). We hypothesized that the discrepancy was due to the choroid’s capacity for autoregulation. The choroid vein wall was thin, the lumen was big, the base was undeveloped, and there was no hemodynamically sensitive venous flap structure. In this study, we measured the thickness *in vivo* instead of sampling the animals after space flight. Consequently, choroid thickness may be influenced by the animal’s living environment during sampling.

Furthermore, OCTA offers detailed, non-invasive, and depth-resolved imaging of the choroidal microvasculature (Laíns et al., 2021). OCTA was also used for dynamic evaluation of the density of rat choroidal capillary in each group, and the results revealed that choroidal capillary density decreased as tail suspension time was increased (Figures 4C, D). Possible and non-exclusive pathophysiological mechanisms for decreased vessel diameter in microgravity include vascular compression due to optic disc/peripapillary swelling and vessel obscuration due to subclinical edema and tissue compression of the various ocular compartments. Venous pooling in the cephalad region, recently demonstrated for the internal jugular vein, may contribute to SANS and loss of blood volume in retinal vessels (Marshall-Goebel et al., 2019; Taibbi et al., 2021). The decrease of choroidal vascular density may be caused by head venous congestion, increased vortex venous pressure, choroidal congestion and dilation, stagnant blood flow, and choroidal capillaries ischemia and hypoxia under a microgravity environment.

Vascular endothelial cells are sensitive to gravity variations. Many studies demonstrated that simulated microgravity leads to apoptosis and autophagy of vascular endothelial cells and influence cytoskeletal, extracellular matrix genes and cell proliferation (Carlsson et al., 2003; Cotrupi et al., 2005). Our results showed aggregation of red blood cells, apoptosis of vascular endothelial cells, enlargement of the mitochondria, and autophagy were seen by electron microscopy in the choroidal capillaries of rats in the TS group with prolonged tail suspension time (Figure 5). Simulated weightlessness can induce ultrastructural changes in choroidal vascular endothelial cells, including cell contraction, chromatin enrichment and marginalization, mitochondrial cavitation, and apoptotic bodies, according to *in vitro* studies (Zhao et al., 2021). Therefore, microgravity can cause endothelial injury of choroid vessels. The difference is that our experiment has confirmed that the head shift of body fluids in a microgravity environment and the choroid vascular endothelial cells injury caused by eye congestion are closer to the real space environment. The retinal pigment epithelium forms the outer blood-retinal barrier, constituting the eye’s microcirculation barrier. The RPE cells’ survival is crucial for maintaining the normal function of the retinal nerve’s upper cortical and lower chorionic capillaries, providing the retina’s structural base and having multiple functions of nutrient exchange and visual cycle maintenance. Therefore, damage to RPE cells will affect the entire optical system. It has been confirmed that ARPE-19 is damaged in a microgravity environment (Corydon et al., 2016; Nguyen et al., 2023). In this study, retinal pigment epithelium cells were swollen and damaged in rats in the tail suspension group. Mitochondrial damage is an important mechanism of neurodegenerative diseases (Fukui and Moraes, 2008). Through the above results, it can be directly confirmed that a microgravity environment can lead to microcirculation disturbance and damage to choroidal vascular endothelial cells and retinal pigment epithelial cells.

As we all know, ischemia, hypoxia, inflammation, and other conditions can cause compromised function of cells, increase permeability and disrupt the integrity of vessel walls, and destroy the blood-retina barrier. Cx43, ZO-1, and occludin are important for maintaining the junction between endothelial cells (Tien et al., 2013). The change in Cx43 level can affect the ZO-1 and occludin expression and regulate the tight junction function, and the decrease of ZO-1 and occludin is the main cause of the breakdown of the blood-retinal barrier (Bhowmick et al., 2019). In this study, the simulated microgravity environment can impede eye microcirculation, reducing the Cx43, ZO-1, and occludin expression.

In the study, we demonstrated that impaired choroid microcirculation can lead to damage to the rats’ retinas mainly at outer layers in tail-suspended simulated weightlessness. This finding was supported by our several observations: 1) typical thinning occurred at the outer layers; 2) the negative wave of ERG (dark-adaptation 3.0 response and light-adaptation 3.0 response) called a wave, reflecting the light absorption activity of the photoreceptors, depressed significantly; 3) the prominent apoptotic cells were largely concentrated at the outer layers. So, why was the outer retina extremely vulnerable to choroidal microcirculation disorders? Primarily, the outer layer of retina is mainly composed of photoreceptor cells, which exhibit vigorous energy metabolism activity in the outer retina and constantly produce significant metabolic wastes and mitochondrial damage. The choroid microvascular system maintains the neuroretina’s visual function by delivering nutrients and oxygen to the retinal cells while eliminating tissue waste. Research has demonstrated that skeletal muscle is sensitive to a decrease in the capillary flow of more than 10%, leading to a sharp decline in muscle performance (Delp et al., 2000). Consequently, our results revealed that the choroidal microcirculation impairment in simulated weightlessness for 5 weeks coincided with the decreased function of retinal photoreceptor cells (dark-adaptation 3.0 response and light-adaptation 3.0 response). Second, photoreceptors are fragile in some way, requiring high energy and nutrients to maintain visual circulatory function. The disturbance of choroidal microcirculation can lead to limited nutrient supply and restricted resistance ability to adverse environments. Finally, the protective threshold of cellular stress response is limited, and sophisticated cellular metabolism pathways are involved, which results in infaust resistance ability (He et al., 2021). It is well known that harmful stress induces apoptosis through the mitochondrial and Bax pathway (Kowluru, 2005; Yan et al., 2021). Western blotting was conducted to verify the BaX and caspase-3 expression related to apoptosis regulation. The results showed that BaX and caspase-3 expression increased, whereas Bcl-2 expression decreased in the TS group. LC3B is a subtype of autophagosome marker gene LC3, and its content increases with more autophagic vesicles, thus serving as a diagnostic indicator for autophagy. Our observations reveal that LC3B expression increases with longer tail suspension, consistent with electron microscopy morphology results (Hwang et al., 2022).

In summary, in our study, the head-down fluid transfer redistribution played a leading role in developing retinal degenerative diseases in a simulated microgravity environment. Under simulated microgravity conditions, fluid transport to the head affects choroidal microvasculature and causes endothelial cell injury. Reduced endothelial cell adhesion impairs the outer blood-retina barrier function, the outer blood-retinal nutrition supply, and the nutrition metabolism of the ONL, causing a loss in retinal function, ONL damage, apoptosis, and degenerative changes. However, the specific mechanism of vascular endothelial cell injury caused by retinal choroidal blood flow change in a microgravity environment is unclear, and further experimental studies will be conducted.

## Data Availability

The original contributions presented in the study are included in the article material, further inquiries can be directed to the corresponding authors.

## References

[B1] Bhowmick S. D'Mello V. Caruso D. Wallerstein A. Abdul-Muneer P. M. (2019). Impairment of pericyte-endothelium crosstalk leads to blood-brain barrier dysfunction following traumatic brain injury. Exp. Neurol. 317, 260–270. 10.1016/j.expneurol.2019.03.014 30926390

[B2] Bimpong-Buta N. Y. Jirak P. Wernly B. Lichtenauer M. Masyuk M. Muessig J. M. (2018). Analysis of human microcirculation in weightlessness: Study protocol and pre-study experiments. Clin. Hemorheol. Microcirc. 70 (1), 119–127. 10.3233/CH-170366 29710687

[B3] Bimpong-Buta N. Y. Muessig J. M. Knost T. Masyuk M. Binneboessel S. Nia A. M. (2020). Comprehensive analysis of macrocirculation and microcirculation in microgravity during parabolic flights. Front. Physiol. 11, 960. 10.3389/fphys.2020.00960 32903511 PMC7438475

[B4] Carlsson S. I. Bertilaccio M. T. Ballabio E. Maier J. A. (2003). Endothelial stress by gravitational unloading: Effects on cell growth and cytoskeletal organization. Biochim. Biophys. Acta 1642 (3), 173–179. 10.1016/j.bbamcr.2003.08.003 14572900

[B5] Corydon T. J. Mann V. Slumstrup L. Kopp S. Sahana J. Askou A. L. (2016). Reduced expression of cytoskeletal and extracellular matrix genes in human adult retinal pigment epithelium cells exposed to simulated microgravity. Cell Physiol. Biochem. 40 (1-2), 1–17. 10.1159/000452520 27842307

[B6] Cotrupi S. Ranzani D. Maier J. A. (2005). Impact of modeled microgravity on microvascular endothelial cells. Biochim. Biophys. Acta 1746 (2), 163–168. 10.1016/j.bbamcr.2005.10.002 16297993

[B7] Delp M. D. Colleran P. N. Wilkerson M. K. McCurdy M. R. Muller-Delp J. (2000). Structural and functional remodeling of skeletal muscle microvasculature is induced by simulated microgravity. Am. J. Physiol. Heart Circ. Physiol. 278 (6), H1866–H1873. 10.1152/ajpheart.2000.278.6.H1866 10843883

[B8] Dickerson B. L. Sowinski R. Kreider R. B. Wu G. (2023). Impacts of microgravity on amino acid metabolism during spaceflight. Exp. Biol. Med. (Maywood). 248, 380–393. 10.1177/15353702221139189 36775855 PMC10281620

[B9] Fukui H. Moraes C. T. (2008). The mitochondrial impairment, oxidative stress and neurodegeneration connection: Reality or just an attractive hypothesis? Trends Neurosci. 31 (5), 251–256. 10.1016/j.tins.2008.02.008 18403030 PMC2731695

[B10] He M. Long P. Chen T. Li K. Wei D. Zhang Y. (2021). ALDH2/SIRT1 contributes to type 1 and type 2 diabetes-induced retinopathy through depressing oxidative stress. Oxid. Med. Cell Longev. 2021, 1641717. 10.1155/2021/1641717 34725563 PMC8557042

[B11] Horie K. Kudo T. Yoshinaga R. Akiyama N. Sasanuma H. Kobayashi T. J. (2018). Long-term hindlimb unloading causes a preferential reduction of medullary thymic epithelial cells expressing autoimmune regulator (Aire). Biochem. Biophys. Res. Commun. 501 (3), 745–750. 10.1016/j.bbrc.2018.05.060 29753741

[B12] Hu N. F. Chang H. Du B. Zhang Q. W. Arfat Y. Dang K. (2017). Tetramethylpyrazine ameliorated disuse-induced gastrocnemius muscle atrophy in hindlimb unloading rats through suppression of Ca2+/ROS-mediated apoptosis. Appl. Physiol. Nutr. Metab. 42 (2), 117–127. 10.1139/apnm-2016-0363 28056188

[B13] Huang A. S. Stenger M. B. Macias B. R. (2019). Gravitational influence on intraocular pressure: Implications for spaceflight and disease. J. Glaucoma 28 (8), 756–764. 10.1097/IJG.0000000000001293 31162175 PMC6786882

[B14] Hwang H. J. Ha H. Lee B. S. Kim B. H. Song H. K. Kim Y. K. (2022). LC3B is an RNA-binding protein to trigger rapid mRNA degradation during autophagy. Nat. Commun. 13 (1), 1436. 10.1038/s41467-022-29139-1 35302060 PMC8931120

[B15] Jung C. Jung F. Kelm M. (2016). The microcirculation in hypoxia: The center of the battlefield for oxygen. Clin. Hemorheol. Microcirc. 63 (3), 169–172. 10.3233/CH-1663301 27567802

[B16] Kamata Y. Hara N. Satou T. Niida T. Mukuno K. (2022). Investigation of the pathophysiology of the retina and choroid in Parkinson's disease by optical coherence tomography. Int. Ophthalmol. 42 (5), 1437–1445. 10.1007/s10792-021-02133-0 34859311 PMC9122847

[B17] Khossravi E. A. Hargens A. R. (2021). Visual disturbances during prolonged space missions. Curr. Opin. Ophthalmol. 32 (1), 69–73. 10.1097/ICU.0000000000000724 33196542

[B18] Kowluru R. A. (2005). Diabetic retinopathy: Mitochondrial dysfunction and retinal capillary cell death. Antioxid. Redox Signal 7 (11-12), 1581–1587. 10.1089/ars.2005.7.1581 16356121

[B19] Laíns I. Wang J. C. Cui Y. Katz R. Vingopoulos F. Staurenghi G. (2021). Retinal applications of swept source optical coherence tomography (OCT) and optical coherence tomography angiography (OCTA). Prog. Retin Eye Res. 84, 100951. 10.1016/j.preteyeres.2021.100951 33516833

[B20] Li S. Song Q. Wu B. Kan G. Wang F. Yang J. (2022). Structural damage to the rat eye following long-term simulated weightlessness. Exp. Eye Res. 223, 109200. 10.1016/j.exer.2022.109200 35932903

[B21] Mader T. H. Gibson C. R. Barratt M. R. Miller N. R. Subramanian P. S. Killer H. E. (2020). Persistent globe flattening in astronauts following long-duration spaceflight. Neuroophthalmology 45 (1), 29–35. 10.1080/01658107.2020.1791189 33762785 PMC7946045

[B22] Mader T. H. Gibson C. R. Caputo M. Hunter N. Taylor G. Charles J. (1993). Intraocular pressure and retinal vascular changes during transient exposure to microgravity. Am. J. Ophthalmol. 115 (3), 347–350. 10.1016/s0002-9394(14)73586-x 8442494

[B23] Mader T. H. Gibson C. R. Miller N. R. Subramanian P. S. Patel N. B. Lee A. G. (2019). An overview of spaceflight-associated neuro-ocular syndrome (SANS). Neurol. India 67 (Suppl. ment), S206–S211. 10.4103/0028-3886.259126 31134911

[B24] Mao X. W. Boerma M. Rodriguez D. Campbell-Beachler M. Jones T. Stanbouly S. (2019). Combined effects of low-dose proton radiation and simulated microgravity on the mouse retina and the hematopoietic system. Radiat. Res. 192 (3), 241–250. 10.1667/RR15219.1 30430917 PMC6820356

[B25] Marshall-Goebel K. Laurie S. S. Alferova I. V. Arbeille P. Auñón-Chancellor S. M. Ebert D. J. (2019). Assessment of jugular venous blood flow stasis and thrombosis during spaceflight. JAMA Netw. Open 2 (11), e1915011. 10.1001/jamanetworkopen.2019.15011 31722025 PMC6902784

[B26] Martin Paez Y. Mudie L. I. Subramanian P. S. (2020). Spaceflight associated neuro-ocular syndrome (SANS): A systematic review and future directions. Eye Brain 12, 105–117. 10.2147/EB.S234076 33117025 PMC7585261

[B27] Nelson E. S. Mulugeta L. Myers J. G. (2014). Microgravity-induced fluid shift and ophthalmic changes. Life (Basel) 4 (4), 621–665. 10.3390/life4040621 25387162 PMC4284461

[B28] Nguyen H. P. Shin S. Shin K. J. Tran P. H. Park H. De Tran Q. (2023). Protective effect of TPP-Niacin on microgravity-induced oxidative stress and mitochondrial dysfunction of retinal epithelial cells. Biochim. Biophys. Acta Mol. Cell Res. 1870 (1), 119384. 10.1016/j.bbamcr.2022.119384 36302465

[B29] Overbey E. G. da Silveira W. A. Stanbouly S. Nishiyama N. C. Roque-Torres G. D. Pecaut M. J. (2019). Spaceflight influences gene expression, photoreceptor integrity, and oxidative stress-related damage in the murine retina. Sci. Rep. 9 (1), 13304. 10.1038/s41598-019-49453-x 31527661 PMC6746706

[B30] Prasad B. Grimm D. Strauch S. M. Erzinger G. S. Corydon T. J. Lebert M. (2020). Influence of microgravity on apoptosis in cells, tissues, and other systems *in vivo* and *in vitro* . Int. J. Mol. Sci. 21 (24), 9373. 10.3390/ijms21249373 33317046 PMC7764784

[B31] Reiner A. Fitzgerald M. E. C. Del Mar N. Li C. (2018). Neural control of choroidal blood flow. Prog. Retin Eye Res. 64, 96–130. 10.1016/j.preteyeres.2017.12.001 29229444 PMC5971129

[B32] Roberts D. R. Albrecht M. H. Collins H. R. Asemani D. Chatterjee A. R. Spampinato M. V. (2017). Effects of spaceflight onAstronaut brain structure as indicated on MRI. New Engl. J. Med. 377, 1746–1753. 10.1056/NEJMoa1705129 29091569

[B33] Stein T. P. (2013). Weight, muscle and bone loss during space flight: Another perspective. Eur. J. Appl. Physiol. 113, 2171–2181. 10.1007/s00421-012-2548-9 23192310

[B34] Sun Y. Kuang Y. Zuo Z. (2021). The emerging role of macrophages in immune system dysfunction under real and simulated microgravity conditions. Int. J. Mol. Sci. 22 (5), 2333. 10.3390/ijms22052333 33652750 PMC7956436

[B35] Taibbi G. Young M. Vyas R. J. Murray M. C. Lim S. Predovic M. (2021). Opposite response of blood vessels in the retina to 6° head-down tilt and long-duration microgravity. NPJ Microgravity 7 (1), 38. 10.1038/s41526-021-00165-5 34650071 PMC8516890

[B36] Tan B. Barathi V. A. Lin E. Ho C. Gan A. Yao X. (2020). Longitudinal structural and microvascular observation in RCS rat eyes using optical coherence tomography angiography. Invest. Ophthalmol. Vis. Sci. 61 (6), 54. 10.1167/iovs.61.6.54 PMC7415900 32579681

[B37] Tien T. Barrette K. F. Chronopoulos A. Roy S. (2013). Effects of high glucose-induced Cx43 downregulation on occludin and ZO-1 expression and tight junction barrier function in retinal endothelial cells. Invest. Ophthalmol. Vis. Sci. 54 (10), 6518–6525. 10.1167/iovs.13-11763 24008412 PMC3790390

[B38] Vernice N. A. Meydan C. Afshinnekoo E. Mason C. E. (2020). Long-term spaceflight and the cardiovascular system. Precis. Clin. Med. 3, 284–291. 10.1093/pcmedi/pbaa022 33391848 PMC7757439

[B39] Wise K. C. Manna S. K. Yamauchi K. Ramesh V. Wilson B. L. Thomas R. L. (2005). Activation of nuclear transcription factor-kappaB in mouse brain induced by a simulated microgravity environment. Vitro Cell Dev. Biol. Anim. 41 (3-4), 118–123. 10.1290/0501006.1 16029073

[B40] Wu H. Zhang G. Shen M. Xu R. Wang P. Guan Z. (2021). Assessment of choroidal vascularity and choriocapillaris blood perfusion in anisomyopic adults by SS-OCT/OCTA. Invest. Ophthalmol. Vis. Sci. 62 (1), 8. 10.1167/iovs.62.1.8 PMC7797932 33393974

[B41] Yan Y. Wang Y. Ding J. Lu L. Ke G. J. Dong K. (2021). TRPML1 inhibited photoreceptor apoptosis and protected the retina by activation of autophagy in experimental retinal detachment. Ophthalmic Res. 64 (4), 587–594. 10.1159/000512104 33027790

[B42] Yang J. W. Song Q. Y. Zhang M. X. Ai J. L. Wang F. Kan G. H. (2022). Spaceflight-associated neuro-ocular syndrome: A review of potential pathogenesis and intervention. Int. J. Ophthalmol. 15 (2), 336–341. 10.18240/ijo.2022.02.21 35186696 PMC8818462

[B43] Yatagai F. Honma M. Dohmae N. Ishioka N. (2019). Biological effects of space environmental factors: A possible interaction between space radiation and microgravity. Life Sci. Space Res. (Amst). 20, 113–123. 10.1016/j.lssr.2018.10.004 30797428

[B44] Zhang L. F. Hargens A. R. (2018). Spaceflight-induced intracranial hypertension and visual impairment: Pathophysiology and countermeasures. Physiol. Rev. 98 (1), 59–87. 10.1152/physrev.00017.2016 29167331

[B45] Zhang Y. Richards J. T. Hellein J. L. Johnson C. M. Woodall J. Sorenson T. (2022). NASA's ground-based microgravity simulation facility. Methods Mol. Biol. 2368, 281–299. 10.1007/978-1-0716-1677-2_18 34647262

[B46] Zhao H. Shi Y. Qiu C. Zhao J. Gong Y. Nie C. (2021). Effects of simulated microgravity on ultrastructure and apoptosis of choroidal vascular endothelial cells. Front. Physiol. 11, 577325. 10.3389/fphys.2020.577325 33536932 PMC7848211

[B47] Zong B. Wang Y. Wang J. Zhang P. Kan G. Li M. (2022). Effects of long-term simulated microgravity on liver metabolism in rhesus macaques. FASEB J. 36 (10), e22536. 10.1096/fj.202200544RR 36070186

